# *Arabidopsis thaliana* alternative dehydrogenases: a potential therapy for mitochondrial complex I deficiency? Perspectives and pitfalls

**DOI:** 10.1186/s13023-019-1185-3

**Published:** 2019-10-29

**Authors:** Alessia Catania, Arcangela Iuso, Juliette Bouchereau, Laura S. Kremer, Marina Paviolo, Caterina Terrile, Paule Bénit, Allan G. Rasmusson, Thomas Schwarzmayr, Valeria Tiranti, Pierre Rustin, Malgorzata Rak, Holger Prokisch, Manuel Schiff

**Affiliations:** 1 UMR1141, PROTECT, INSERM, Université de Paris, Paris, France; 2 Unit of Medical Genetics and Neurogenetics, Fondazione IRCCS Istituto Neurologico Carlo Besta, Milan, Italy; 3 Institute of Human Genetics, Helmholtz Zentrum München, German Research Center for Environmental Health, Neuherberg, Germany; 4 Institute of Human Genetics, Technische Universität München, Munich, Germany; 5 Reference Center for Inborn Errors of Metabolism, Hôpital Universitaire Robert Debré, APHP, Paris, France; 6 Department of Biology, Lund University, Biology building A, Sölvegatan 35, SE-22362 Lund, Sweden; 7 Dr. von Hauner Children’s Hospital, Department of Pediatrics, University Hospital, Ludwig-Maximilians-Universität (LMU), Munich, Germany

**Keywords:** Mitochondria, Mitochondrial diseases, Complex I, Alternative dehydrogenases, *Arabidopsis thaliana*, AtNDA2

## Abstract

**Background:**

Complex I (CI or NADH:ubiquinone oxidoreductase) deficiency is the most frequent cause of mitochondrial respiratory chain defect. Successful attempts to rescue CI function by introducing an exogenous NADH dehydrogenase, such as the NDI1 from *Saccharomyces cerevisiae* (ScNDI1), have been reported although with drawbacks related to competition with CI. In contrast to ScNDI1, which is permanently active in yeast naturally devoid of CI, plant alternative NADH dehydrogenases (NDH-2) support the oxidation of NADH only when the CI is metabolically inactive and conceivably when the concentration of matrix NADH exceeds a certain threshold. We therefore explored the feasibility of CI rescue by NDH-2 from *Arabidopsis thaliana* (At) in human CI defective fibroblasts.

**Results:**

We showed that, other than ScNDI1, two different NDH-2 (AtNDA2 and AtNDB4) targeted to the mitochondria were able to rescue CI deficiency and decrease oxidative stress as indicated by a normalization of SOD activity in human CI-defective fibroblasts. We further demonstrated that when expressed in human control fibroblasts, AtNDA2 shows an affinity for NADH oxidation similar to that of CI, thus competing with CI for the oxidation of NADH as opposed to our initial hypothesis. This competition reduced the amount of ATP produced per oxygen atom reduced to water by half in control cells.

**Conclusions:**

In conclusion, despite their promising potential to rescue CI defects, due to a possible competition with remaining CI activity, plant NDH-2 should be regarded with caution as potential therapeutic tools for human mitochondrial diseases.

**Electronic supplementary material:**

The online version of this article (10.1186/s13023-019-1185-3) contains supplementary material, which is available to authorized users.

## Introduction

Human NADH:ubiquinone oxidoreductase or complex I (CI) is the largest complex of the respiratory chain, with a mass of 980 kDa and 44 different subunits encoded by both mitochondrial and nuclear genomes [1].

CI catalyzes the consecutive transfer of two electrons, one per time, to a ubiquinone pool for each molecule of NADH oxidized. NADH oxidizing activity of CI is tightly controlling intra-mitochondrial metabolism, and electron transfer is coupled to both heat and ATP generation. Electron transfer is associated with the pumping of 4H+ across the inner mitochondrial membrane, which sustains part of the mitochondrial membrane potential [2]. The 44 subunits are arranged in three functional modules: the N module involved in oxidizing NADH, the Q module involved in reducing ubiquinone, and the P module dedicating proton translocation [3]. A number of mutations in nuclear and in mitochondrial genes coding for many of the 44 subunits, as well as in genes coding for assembly or regulatory factors, have been shown to result in CI deficiency [4]. Therefore, CI deficiency can result in a combination of abnormalities: impaired oxidation of NADH to NAD+, which alters the NADH/NAD+ ratio and leads to intra-mitochondrial metabolic disequilibrium and ultimately to lactic accumulation, release of electrons that are not correctly channeled to the ubiquinone subsequently generating radical oxygen species (ROS), and loss of proton pumping activity, which reduces mitochondrial potential, hence lowering ATP synthesis.

**Fig. 1:**
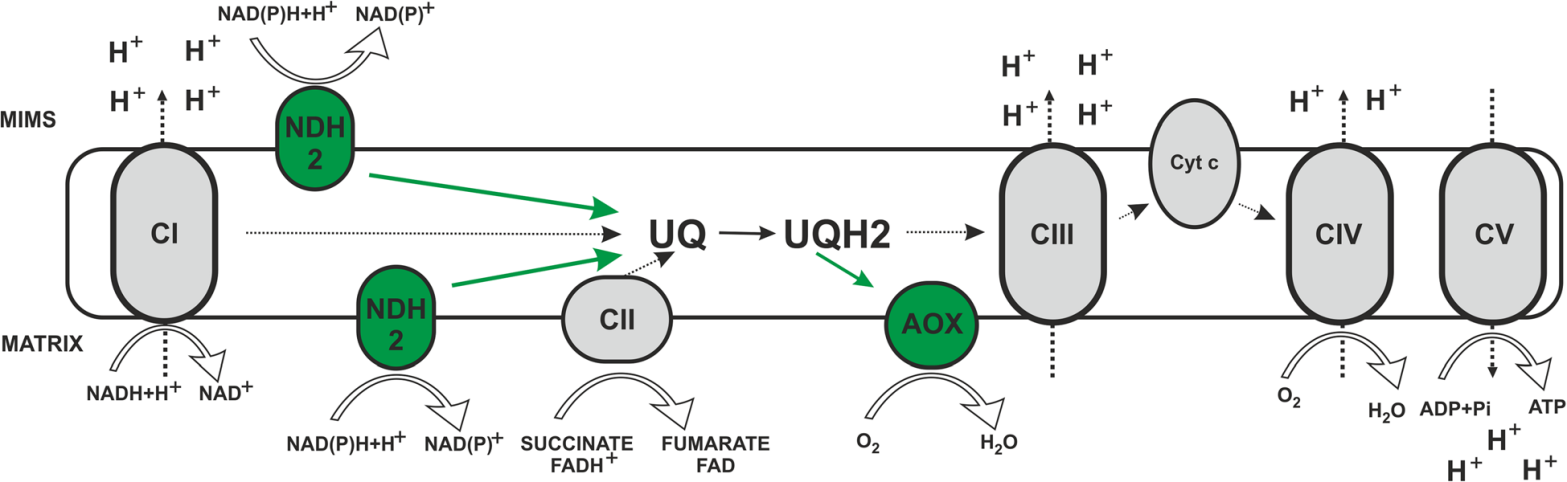
Mitochondrial respiratory chain and alternative enzymes. Schematic representation of the canonical mitochondrial respiratory chain (in black and white) characterized by four multi-subunit complexes (Complex I, Complex II, Complex III and Complex IV) and two intermediary substrates (ubiquinone and cytochrome *c*) generating an electrochemical gradient through the inner mitochondrial membrane. Protons flow back to the matrix via Complex V to produce ATP. The figure also illustrates alternative pathways of NAD(P)H and ubiquinol oxidation (in green) represented by alternative dehydrogenases (NDH2) and alternative oxidases (AOX), respectively. CI to CV, complexes I to V; UQ, ubiquinone; UQH2, ubiquinol; Cyt c, cytochrome *c*; MIMS, mitochondrial intermembrane space

In microbes, fungi, plants, and also in some metazoan phyla (but not in arthropods or vertebrates), two key steps of the mitochondrial respiratory chain, namely ubiquinone reduction and ubiquinol oxidation, differ from mammals as they involve bypassing enzymes: alternative NADH dehydrogenases (NDH-2) and alternative oxidases (AOXs). NDH-2 can functionally replace NADH oxidizing activity of CI, transferring electrons from NADH directly to ubiquinone, while AOXs can be a functional substitute of complexes III and IV (AOXs being able to transfer electrons from a ubiquinol pool directly to oxygen, see Fig. 1) [5].

These alternative enzymes possess some key properties that distinguish them from other mitochondrial complexes: they are single or oligo subunit, non-proton pumping enzymes, as the energy they convey during their activation does not support mitochondrial potential; they are not inhibited by cytochrome pathway inhibitors (e.g. rotenone and cyanide) and, in contrast to other mitochondrial complexes, they are not transmembrane proteins but are associated to either the inner or the outer surface of the inner mitochondrial membrane [6, 7].

In *Saccharomyces cerevisiae* CI is absent and replaced by the ScNDI1 protein. In an attempt to rescue CI deficiency, Yagi and collaborators introduced this type II NAD(P) H dehydrogenase from yeast, ScNDI1, into mammalian cells with impaired CI. This resulted in the recovery of NADH oxidation and reduction of ROS production in a variety of CI defective cell cultures harbouring mutations in either *ND4*, *ND5* or *NDUFA1* [8, 9]. Cells with CI deficiency acquired the ability to grow in a non-fermentable medium, such as galactose, upon transfection with ScNDI1. Moreover, ScNDI1 has proven beneficial in fly models of CI deficiency [10].

This concept was further developed in gene therapy approaches in mice and rats. Bypassing CI by expression of ScNDI1 was demonstrated to be well tolerated. Furthermore, ScNDI1 protected rat neurons against the CI specific inhibitor rotenone, rescued CI deficiency [11–13], and showed potential therapeutic effects in a mouse model of Parkinson disease [14].

However, when introduced into control HEK293 cells, ScNDI1 caused a decrease in the amount of ATP produced *per* oxygen reduced (P/O ratio) for the CI-dependent respiration from a value of 2.5 to 1.8 [15], showing that ScNDI1 is active even in the presence of a fully functional CI, therefore competing with CI for the oxidation of NADH. Such competition could compromise energy production and lower mitochondrial potential, thus potentially leading to unpredictable metabolic consequences.

Unlike *Saccharomyces cerevisiae*, which lacks CI, many plants have NDH-2, which naturally coexist with CI. They oxidize NADH only in specific physiologic conditions, depending on the nature of the available organic acids, considering that some alternative dehydrogenases from plants were shown to have a 3 to 10 folds higher K_M_ for NADH than plant CI in native conditions [16–18], or hypothetically on the presence of specific matrix compartmentalized NADH pools.

*Arabidopsis thaliana*, in particular, expresses different isoforms of NDH-2 associated either to the inner or to the outer mitochondrial membrane. The intrinsic role of these alternative systems could be to maintain a redox balance and a proper turn-over of mitochondrial metabolism, continuing to oxidize substrates when the metabolic demand is modified. This particularly manifests during daylight exposition of plants, when OXPHOS is inhibited by the extensive mobilization of cytosolic ADP by the photosynthetic process: and indeed, it was shown that activation/expression of NDH-2 takes place in physiologic conditions lowering CI activity [19].

Thus, the plant enzyme is expected to naturally take over for NADH oxidation only when CI is prevented from working, providing a potential mechanism to mitigate the redox imbalance in cells with defective CI, without competing with its endogenous residual activity.

A very similar strategy based on the expression of the tunicate *Ciona intestinalis* alternative oxidase (AOX) has previously been shown to exert beneficial effects in counteracting the consequences of complexes III or IV respiratory chain deficiency in human cells and animal models [20], even though relevant constraints deriving from a profound influence on energy production and other biological processes have been recently reported after transfection in *Drosophila* [5].

Taken together, all these considerations open a way to forecast xenotopic transfection of genes encoding for plant’s NDH-2 as a conceivable treatment for CI deficiency, as these enzymes should be active only when electron transfer from NADH through CI is impaired. Therefore, we evaluated the potential benefit of introducing alternative dehydrogenases AtNDA2 and AtNDB4 from *Arabidopsis thaliana* into a CI defective patient fibroblast cell line carrying a homozygous mutation in *NDUFS4* and compared it to ScNDI1 from *Saccharomyces cerevisiae*. Moreover, we assessed kinetic and biochemical effects of one of these proteins (AtNDA2) in control fibroblasts.

## Materials and methods

### Cell transfection and selection

For the evaluation of the above-described therapeutic strategy on cellular models, we focused on control and CI defective human fibroblasts.

The control fibroblasts (NDHF) were purchased from Lonza (Cat. No. CC-2509). Patients’ fibroblasts were obtained from skin biopsies of patients with signed informed consent. The CI defective cell line (79787) belongs to a patient affected with Leigh syndrome carrying the homozygous frameshift mutation c.462delA (p.Lys154fs) within *NDUFS4*, located in 5q11 and encoding for a CI subunit close to the catalytic region of NADH-quinone oxidoreductase. The mutation is predicted to result in the synthesis of a truncated protein. Indeed, the absence of NDUFS4 protein was previously reported in fibroblasts derived from patients harbouring the same *NDUFS4* homozygous mutation [21].

Skin fibroblast cells were grown in Dulbecco’s modified Eagle’s medium (DMEM) with Glutamax +/− 4.5 g/L Glucose, supplemented with 10% fetal calf serum (FBS), 2.5 mM Pyruvate and maintained in a 5% CO2 incubator at 37 °C. Patients’ fibroblasts were obtained from skin biopsies of patients and signed informed consent. Selective growth of transfected cells was maintained by adding blasticidin 5 μg/ml to DMEM.

**Fig. 2:**
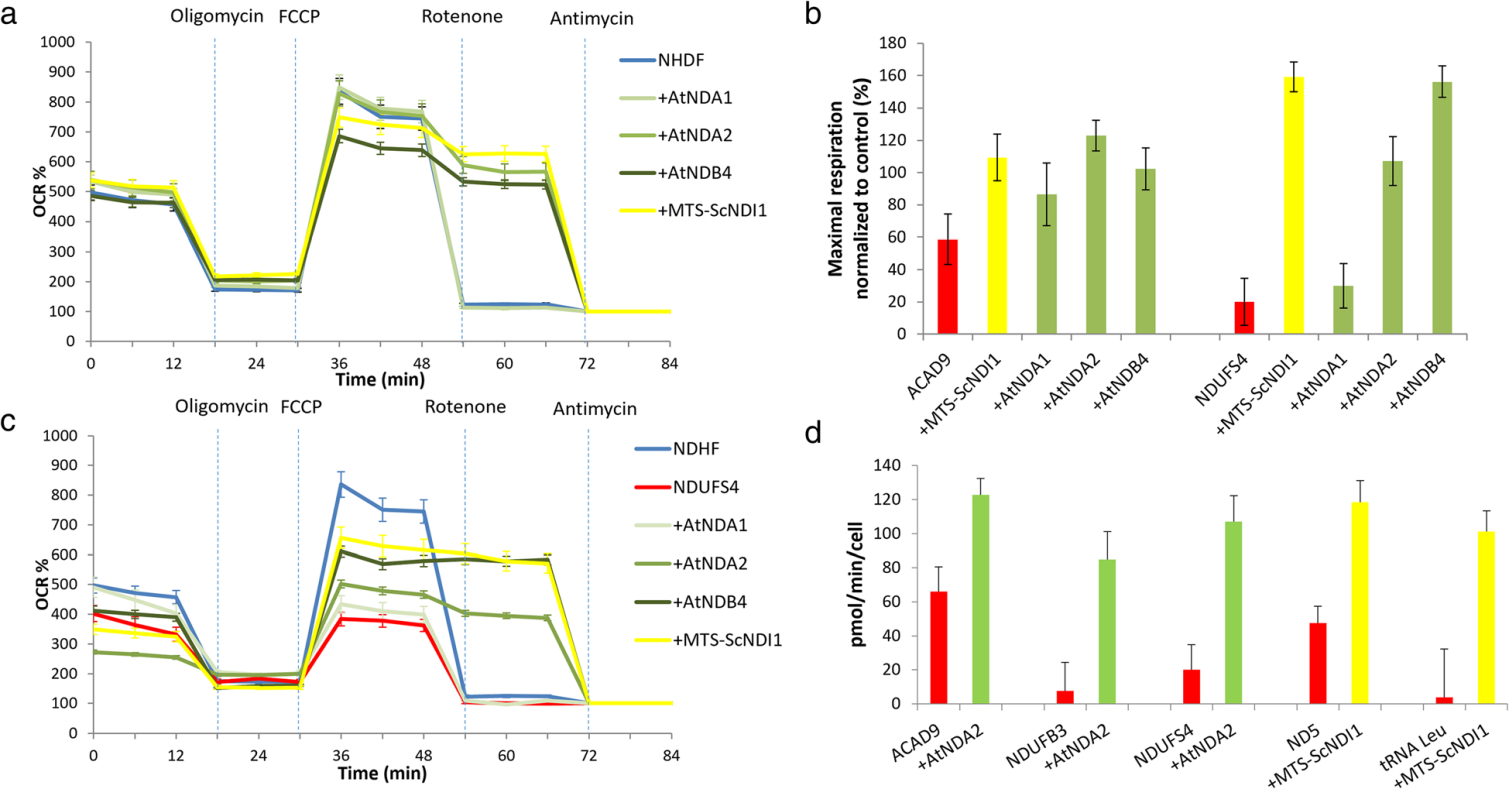
Oxygen consumption analysis: Oxygen consumption was evaluated using the Seahorse XF Analyzer; **a** Oxygen consumption rate (OCR) expressed as percent (%) of rate measurement 13 in control cells (NDHF) and in control cells transduced with alternative dehydrogenases from *A. thaliana* (+AtNDA1, +AtNDA2, +AtNDB4) and yeast (+MTS-ScNDI1); **b** OCR expressed as % of rate measurement 13 in NDHF, in NDUFS4-deficient cells (NDUFS4) and in patient cells transduced with alternative dehydrogenases from *A. thaliana* (+AtNDA1,+AtNDA2, +AtNDB4) and yeast (+MTS-ScNDI1); **c** Maximal respiration rate in CI deficient cells (carrying pathogenic variants in ACAD9 and NDUFS4), before and after transduction with AtNDA1, AtNDA2, AtNDB4 and MTS-ScNDI1. Values were normalized to maximal respiration of untransduced control cells; **d** Oxygen consumption rate (OCR) expressed as pmol O_2_/min/cell in cell lines presenting with CI-defect due to mutations in ACAD9, NDUFB3, NDUFS4, ND5, tRNA^Leu^ before and after transduction with alternative dehydrogenases from *A. thaliana* and yeast. Each cell line was measured at least twice in independent experiments. During experiment, four technical replicates were run for each cell line. Values are expressed as mean ± SD

Control and patient fibroblasts were transfected with constructs containing the four NDH-2 genes of interest (AtNDA1, AtNDA2, AtNDB4 and ScNDI1) fused with human mitochondrial targeting signal (MTS) and a blasticidin resistance sequence (Additional file 1: Supplementary Methods). Transfection has been performed using a lentiviral vector from Invitrogen™ (ViraPower™ HiPerform™) according to Kremer and Prokisch [22]. Assessment of transduction efficacy and selection of transfected cell lines were performed using the results of qPCR (not shown) and oxygen consumption analysis (Fig. 2) as previously described [22].

### Enzymatic activity assay and determination of kinetic parameters

Collection and permeabilization of fibroblasts were performed as previously described [23].

Spectrophotometric analysis of NADH:quinone oxidoreductase specific activity was performed on a Cary 60 spectrophotometer equipped with a18-cell holder maintained at 37 °C.

Measures of NADH:quinone oxidoreductase specific activity were performed in buffer A containing 10 mM KH_2_PO_4_, pH 7.2 and 1 mg/mL BSA at wavelengths of 340 nm–380 nm to assess NADH oxidation using an extinction coefficient of 4.87 as previously described [23, 24].

The sample compartment was kept open in order to allow manual stirring of the cuvette content after each addition. For K_M_ determination, samples (8–20 μL) were added to water, incubated for 1 min before mixing with buffer A. Rotenone (8 μM), KCN (650 μM), DCQ (50 μM) were sequentially added to the cuvettes before starting the reaction with the substrate NADH (at concentrations ranging from 0.3 to 150 μM) and following the reaction kinetics. A comparative assay was performed without rotenone in order to quantify the amount of rotenone resistant NADH:quinone oxidoreductase activity. All the measurements were performed at least in triplicates.

K_M_ and Vmax were estimated using an online available tool (http://www.ic50.tk/K_M_vmax.html) using the Michaelis-Menten model.

Proteins were measured according to Bradford [25].

### Superoxide dismutase (SOD) activity assessment

SOD activity was measured according to Stefan L. Marklund following the described method of pyrogallol auto-oxidation inhibition. One unit of SOD inhibits 50% of pyrogallol autoxidation, measured at 420 nm [26].

### P/O assay

Subconfluent fibroblasts (75 cm^2^ flask) were trypsinized and the pellet was washed once with 1 mL PBS. The oxygen uptake was measured with an optic fiber equipped with an oxygen-sensitive fluorescent terminal sensor (Optode device: FireSting O_2_, Bionef, Paris, France). The optic fiber was fitted to a printed cap ensuring the closure of the quartz-cell yet allowing micro-injections (0.6 mm hole diameter) for concurrent measurement of oxygen uptake with mitochondrial potential (determined by the fluorescence change of 100 nM rhodamine). Cells were added to 750 μL of buffer consisting of 0.25 M sucrose, 15 mM KCl, 30 mM KH_2_PO_4_, 5 mM MgCl_2_, 1 mM EGTA, pH 7.4, followed by the addition of rhodamine (100 nM), BSA 1 mg /ml and 0.01% w/v digitonin. The permeabilized cells were successively added followed by the addition of mitochondrial substrates (6.25 mM glutamate/malate or 6.25 mM succinate) and two consecutive injections of ADP (40 nmol each) to ensure state 3 (phosphorylating) conditions or ATP (40 nmol) in order to estimate ATP recycling due to ATPases activity. The reaction was followed until state 4 (the respiratory rate after all the ADP has been phosphorylated to form ATP) was reached back and maintained. Respiratory rates during state 3 and state 4 were estimated as the speed of oxygen consumption (nmol/min) adjusted to protein concentration (μg) in each cuvette. Respiratory control index was later calculated as the ratio between state 3 to state 4 respiratory rates. P/O values (corresponding to the number of ATP molecules produced for every oxygen atom consumed) were also measured as the ratio between concentration (in nmol) of ADP (or ATP) added to the cuvette and the amount of oxygen atoms (nmol of molecular oxygen*2) consumed during state 3 to state 4 transition. All the assays were repeated at least three times. Protein content was measured according to Bradford [25].

### RNA sequencing

RNA sequencing was performed as described [27]. Briefly, RNA was isolated from whole-cell lysates using the AllPrep RNA Kit (Qiagen) and RNA integrity number (RIN) was determined with the Agilent 2100 BioaAnalyzer (RNA 6000 Nano Kit, Agilent). For library preparation, 1 μg of RNA was poly(A)- selected, fragmented and reverse transcribed with the Elute, Prime and Fragment Mix (Illumina). End repair, A-tailing, adaptor ligation and library enrichment were performed as described in the Low Throughput protocol of the TruSeq Stranded mRNA Sample Prep Guide (Illumina). RNAcDNA libraries were assessed for quality and quantity with the Agilent 2100 BioaAnalyzer and quantity using the Quant-iT PicoGreen dsDNA Assay Kit (Life Technologies). RNA libraries were sequenced as 150 bp paired-end runs on an Illumina HiSeq4000 platform. The STAR aligner* (v 2.4.2a) with modified parameter settings (−-twopassMode = Basic) was used for split-read alignment against the human genome assembly hg19 (GRCh37) and UCSC knownGene annotation. Prior alignment, the reference genome sequence was augmented by two new contigs, one for each plant gene (NDA2 and NDB4, respectively). The nucleotide sequences of these two genes corresponded to the transgenic constructs cloned in the lentiviral vector (see Additional file 1: Supplementary Methods). To quantify the number of reads mapping to annotated genes we used HTseq-count (v0.6.0). FPKM (Fragments *Per* Kilobase of transcript per Million fragments mapped) values were calculated using custom scripts.

### Statistical analysis

All data are expressed as the mean ± SD, and comparisons between groups performed using Student’s *t* test.

## Results

### Proof of concept that NDH-2 dehydrogenases counteract CI deficiency

Preliminary assays on different CI defective fibroblast cell lines had showed the ability of several NDH-2 to rescue respiration defect (Fig. 2b-d). We decided to focus our subsequent analysis on three NDH-2: ScNDI1, the internal NDH-2 of *Saccharomyces cerevisiae*; AtNDB4, an *Arabidopsis thaliana* NDH-2 localized to the external side of inner mitochondrial membrane (IMM); AtNDA2, another *Arabidopsis thaliana* NDH-2 localized to the internal side of IMM.

As expected, transfection of control fibroblasts with AtNDA2, AtNDB4, and ScNDI1 led to rotenone-resistance without any significant effect on overall respiration rate (Fig. 2a). In order to examine the rescuing efficiency of plant NDH-2 in more depth, we chose to focus on fibroblasts carrying a pathogenic homozygous mutation in the nuclear gene *NDUFS4*, as a well-established cellular model of complex I deficiency. Indeed, consequences of deleterious mutations affecting NDUFS4 have been thoroughly studied on several patient cell lines and on whole-body and tissue specific knockout mice [28].

Therefore, we verified and confirmed that besides conferring rotenone resistance, all the aforementioned NDH-2 (ScNDI1, AtNDA2 and AtNDB4) were able to restore respiration when expressed in NDUFS4 deficient fibroblasts, nearly reaching control levels. (Fig. 2c).

### Expression of NDH-2 dehydrogenases does not affect growth of cultured human fibroblasts

ScNDI1, AtNDA2, and AtNDB4 transfected control fibroblasts (NHDF) and CI-defective fibroblasts (NDUFS4) exhibited comparable growth rates when compared to the corresponding untransfected control, both in glucose (4.5 g/L) and glucose-deprived media (not shown).

### At-NDA2 and at-NDB4 rescue NADH:quinone oxidoreductase activity of NDUFS4 mutant fibroblasts

We further confirmed the observed rescue by measuring NADH:quinone oxidoreductase specific activity by spectrophotometry in control cells and *NDUFS4* mutated fibroblasts before and after transfection by ScNDI1, AtNDA2, and AtNDB4, show that all three dehydrogenases were able to rescue the CI defect (Table 1). We could also observe that, while AtNDA2 and AtNDB4 restored CI activity to levels comparable to those observed in control cells, ScNDI1 transfected cells exhibited levels of NADH:quinone oxidoreductase activity much higher than untransfected cells (Table 1).

**Table 1:** NADH:quinone oxidoreductase activity in control and transfected cells

Cell line	NADH:quinone oxidoreductase specific activity w/o rotenone (nmol/min/mg Prot)	NADH:quinone oxidoreductase specific activity with rotenone (nmol/min/mg Prot)	Inhibition (%)
NHDF	7.7 (±0.5)	1.7 (±0.2)	78.3%
79,787	2.5 (±0.2)	1.4 (±0.1)	42.6%
79,787-T-ScNDI1	24.0 (±8.7)	20.7 (±8.3)	13.7%
79,787-T-AtNDB4	11.8 (±2.4)	10.6 (±1.9)	10.0%
79,787-T-ATNDA2	5.3 (±0.9)	5.3 (±0.9)	2.7%

Spectrophotometric assessment of NADH:quinone oxidoreductase specific activity in control cells (NDHF) and *NDUFS4* cell line untransfected (79787) and transfected with alternative NADH oxidases (ScNDI1, AtNDA2 and AtNDB4). Values are expressed as mean ± SD

### SOD activity in NDUFS4 deficient cells

**Fig. 3:**
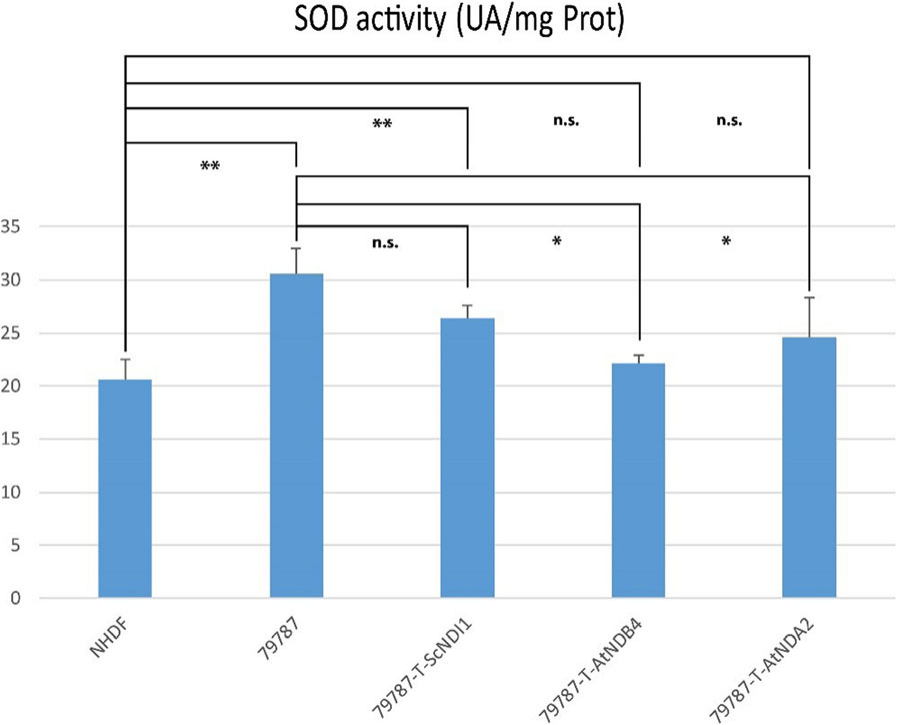
Assessment of SOD activity: Spectrophotometric assessment of SOD activity according to the pyrogallol autoxidation method. *NDUFS4* mutated cell line (79787) displays higher SOD activity when compared to control fibroblasts (NHDF). Transfection with AtNDB4 and AtNDA2 (79787-AtNDB4 and 79,787-AtNDA2) significantly decreases SOD activity, which is nearly restored to normal levels. Values are expressed as means ± SD (ns: not significant; *p < 0.05; **p < 0.01)

As a direct effect of defective CI activity and consequent increase of ROS production, SOD activity is shown to be significantly higher in *NDUFS4* mutated patient’s cell (Fig. 3). Transfection with AtNDA2 and AtNDB4 but not with ScNDI1 was almost able to decrease SOD activity to levels observed in control fibroblasts (Fig. 3).

### AtNDA2 and AtNDB4 expression in control cell lines

We evaluated by RNA sequencing the expression levels of CI subunits, AtNDA2, and AtNDB4 in control cells before and after the stable transduction with the plant genes AtNDA2 and AtNDB4 (RNA sequencing was not performed on NDUFS4 deficient fibroblasts due to scarcity of material). We evaluated the FPKM values for CI subunits before and after transduction. The median FPKM of CI subunits was similar in all cell lines, indicating that the transduction with the plant genes did not affect the expression level of CI subunits (median FPKM between 30 and 35, Table 2). AtNDA2 had an expression level of 25 FPKM, which falls in the range of CI subunit expression, while AtNDB4 had an expression level of 127 FPKM, much higher than the median expression level of CI subunits (Table 2). In *A. thaliana* the endogenous expression of NDA2 and NDB4 is significantly lower than the expression of CI subunits, in all parts of the plant (flower, root, leaf, and fruit). The expression of NDA2 is 10 times lower than the median expression of CI subunits, while NDB4 is almost 500 times lower than the median expression of CI subunits [29] (Additional file 1: Table S1).

**Table 2:**
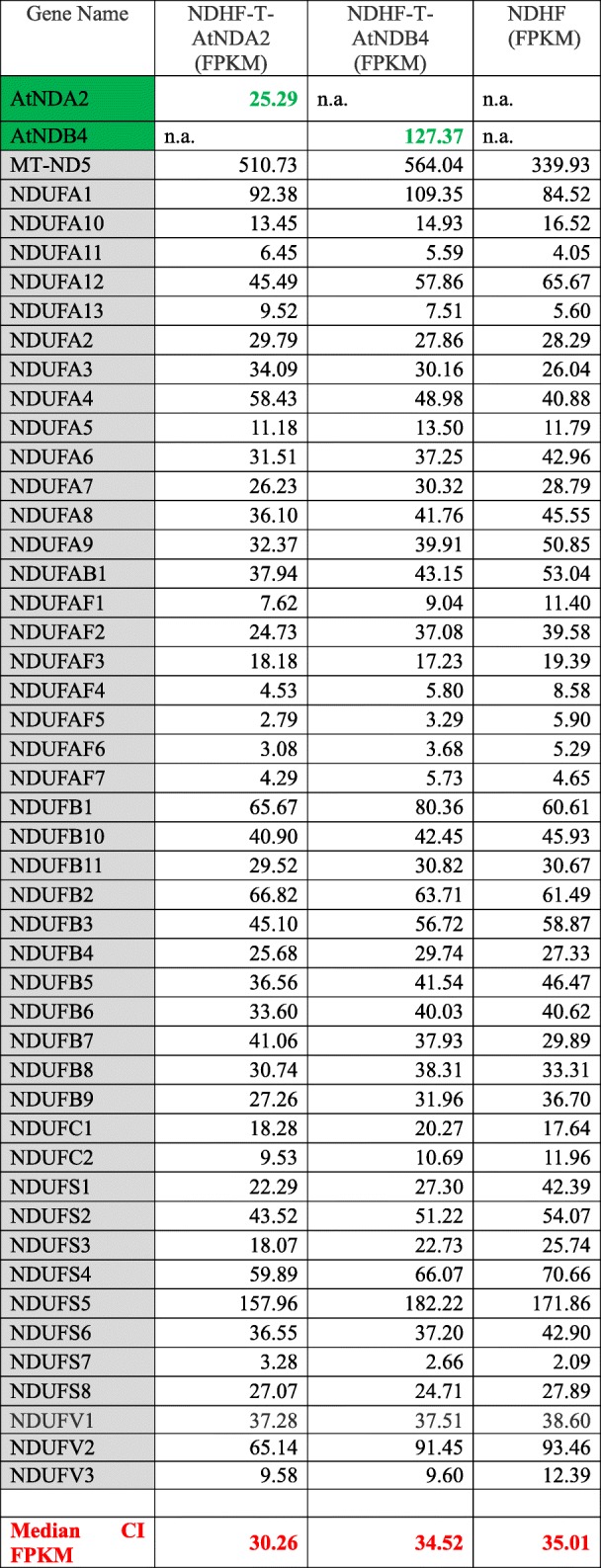
Expression level of AtNDA2, AtNDB4, and NADH:quinone oxidoreductase (CI) subunits in control cell lines (NDHF) before and after transduction with AtNDA2 and AtNDB4

Expression levels are indicated in FPKM. In gray are labelled the NADH:quinone oxidoreductase subunits, in green the two NDH-2 genes. Median FPKM for CI subunits in both cell lines is given in red

### AtNDA2 competes with CI when expressed in human fibroblasts

**Fig. 4:**
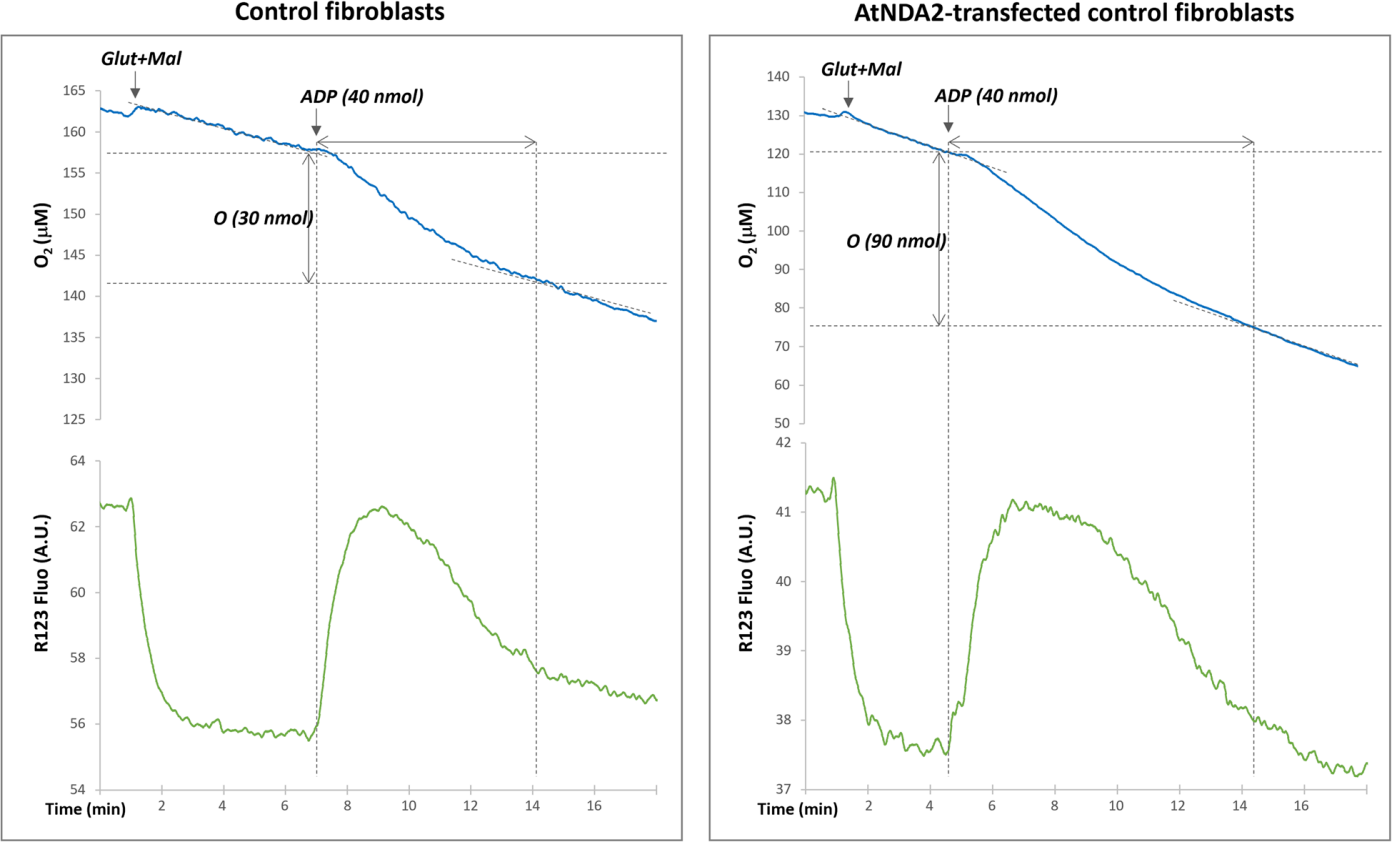
Evaluation of mitochondrial respiration: Mitochondrial membrane potential variations assessed by rhodamine 123 fluorescence and oxygen uptake measured with optode device in digitonine permeabilized fibroblasts (representative graphs for the control fibroblasts - left panel and AtNDA2-transfected control fibroblasts - right panel). The reaction was started by the addition of glutamate/malate, followed by injections of ADP (see text). Note that the amount of oxygen reduced during ADP phosphorylation is significantly higher in AtNDA2 transfected cells comparing to control

**Fig. 5:**
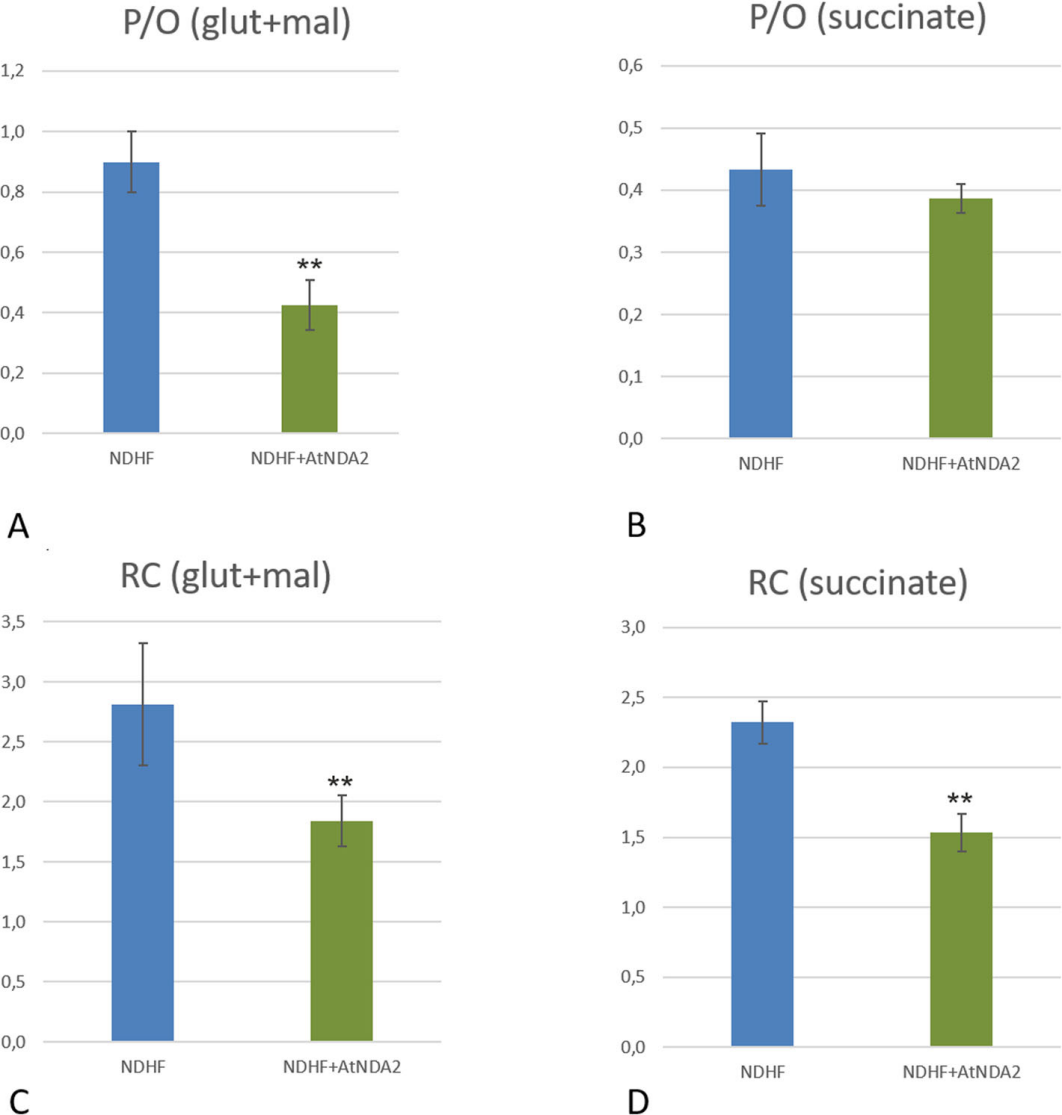
RC (respiratory control) and P/O coupling ratios: Comparison of P/O values (**a** and **b**) and Respiratory Control Index (**c** and **d**) with Glutamate/Malate (**a**,**c**) and Succinate (**b**, **d**) in non-transfected control and AtNDA2 transfected control cells

Thereafter, in view of its expression profile in *A. thaliana* (Add file 1), we selected the AtNDA2 to verify the lack of competition between CI and plant NDH-2 when expressed in control fibroblasts. We first studied the P/O ratio with different substrates, the latter being supposedly decreased if NADH, normally oxidized by the proton-motive CI, is diverted to AtNDA2. P/O calculation is usually performed on isolated mitochondria in order to eliminate cytosolic ATPases activity. ATPases increase ADP recycling, allowing for a continuous stimulation of the mitochondrial ATP synthase and respiration, thus affecting state 4 establishment. However, taking into consideration the scarcity of material and the slow growth rate of fibroblasts, we performed the assays using permeabilized cells. As expected, the observed P/O values were underestimated comparing to the ones measured on purified mitochondria (about 2.5 for NADH related substrates and 1.5 for succinate, respectively)-see Hinkle et al. [30] for a complete review on this topic. Nevertheless, using this approach, we were able to measure the P/O ratio (Fig. 4). Unexpectedly, we evidenced a competition between AtNDA2 and functional mitochondrial respiratory chain CI during glutamate/malate oxidation in the control cell line expressing AtNDA2 (Fig. 4). Transfected cells showed P/O values lowered by half (0.43 ± 0.08) in comparison to untransfected cells (0.9 ± 0.1). Moreover, respiratory control index, calculated as the ratio between state 3 and state 4, which represents a numeric estimation of mitochondrial coupling efficiency, was also clearly lowered in transfected cells under glutamate/malate stimulation (Fig. 5).

A further validation of these results came from P/O data obtained with succinate, the substrate of CII. Indeed, although P/O ratios for succinate were not significantly lower in transfected cells, 0.43 ± 0.05 and 0.39 ± 0.02, respectively, we observed a reduction of respiratory control index in transfected cells (Fig. 5). This is likely due to the metabolic conversion of a fraction of succinate to glutamate, which later enters the oxidation machinery proceeding through CI and AtNDA2.

When ATP instead of ADP was used, we observed only a very low OXPHOS stimulation in both transfected and untransfected cell lines, providing an additional confirmation that our measurments were not significantly affected by ATPase mediated ATP recycling to ADP (not shown).

**Fig. 6:**
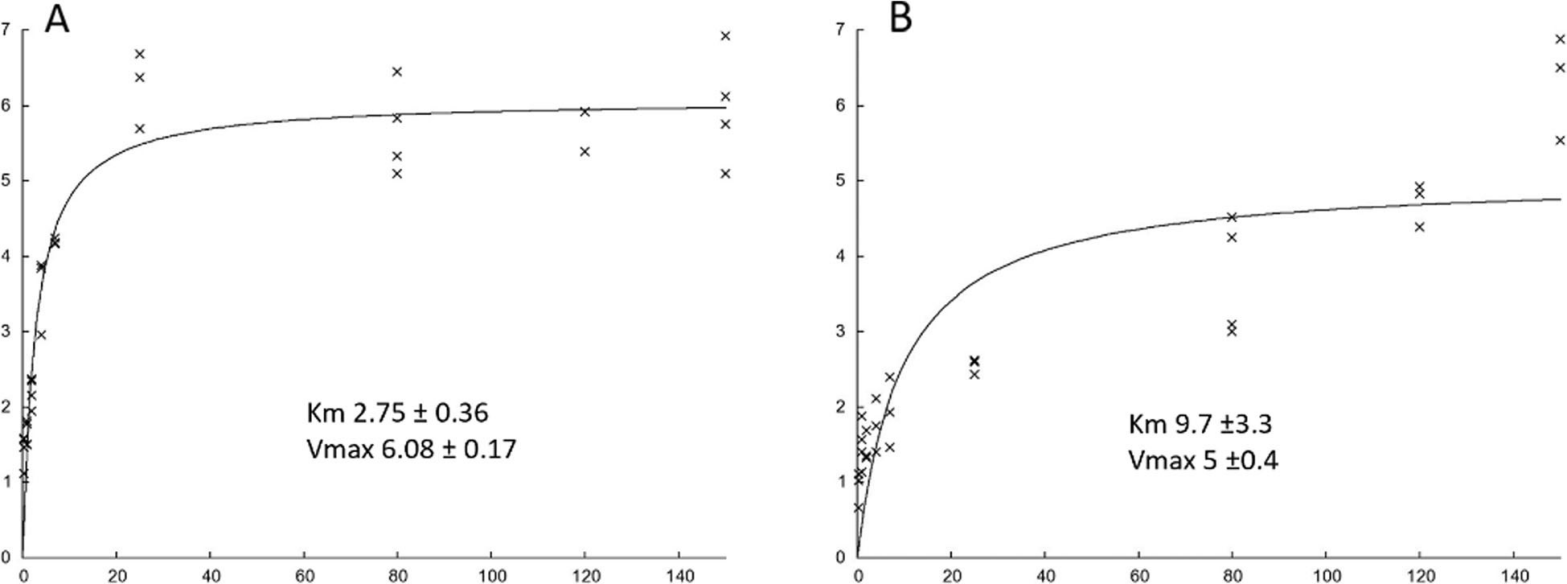
K_M_ evaluation: Plots of NADH:quinone oxidoreductase activity (*y*) as a function of NADH concentration (μM) (*x*): **a** rotenone-sensitive NADH:quinone oxidoreductase activity in control cells; **b** rotenone-resistant NADH:quinone oxidoreductase activity in AtNDA2 transfected control cells

A further evidence of the likely competition between CI and AtNDA2, came from calculation of K_M_ for NADH in both transfected and untransfected cell lines (Fig. 6). CI affinity for NADH was estimated in untransfected control cells considering only rotenone sensitive internal NADH:quinone oxidoreductase activity, while, to evaluate AtNDA2 affinity for NADH, we exclusively analyzed rotenone insensitive activity in AtNDA2-transfected control cells. Our estimation of CI and AtNDA2 K_M_ gave values of 2.7 ± 0.4 μM and 9.7 ± 3.3 μM, respectively. Hence, when transfected in human cells, K_M_ of AtNDA2 for NADH appear to be about only 3 folds higher than K_M_ of CI for NADH, i.e. in the same order of magnitude; this gap is probably insufficient to prevent competition for the substrate within physiological range of NADH concentration inside the mitochondria, therefore indirectly confirming that upon these experimental conditions, it seems likely that CI and AtNDA2 compete for the oxidation of NADH.

## Discussion

CI is the largest complex of the respiratory chain, consisting of 44 different subunits encoded by both nDNA and mtDNA. These subunits are assembled in a precise order by numerous assembly factors [1]. Thus, pathogenic mutations in genes encoding either for structural subunits or for assembly factors, can result in the enzymatic impairment of CI, often with a yet poorly understood tissue-specificity and time-dependency*.* Besides these mechanisms, CI deficiency may arise as a consequence of mutations in genes encoding proteins involved in mitochondrial translation, in iron-sulphur cluster assembly, and mtDNA-depletion associated genes [31, 32]. This could explain why CI deficiency is the far most common finding in mitochondrial disorders.

In terms of therapeutic approach, having a unique treatment applicable to all CI deficiencies, regardless of the genetic cause, would be desirable. A bypassing strategy using alternative dehydrogenase proteins seems to offer such possibility. Indeed the monomeric NADH dehydrogenase of yeast, ScNDI1, inserted in CI-deficient cells by Yagi and colleagues [14], presented an apparent beneficial effect on several experimental models in vitro and in vivo [8–13]. Nevertheless, it lowered P/O values for CI-dependent oxidation of NADH indicating reduced ATP synthesis upon ScNDI1 transfection in control mammalian cells [14] raising questions about its feasibility as treatment for patients with impaired but residual CI activity, where prevalence of ScNDI1 over residual CI activity could worsen metabolic disturbances and lower OXPHOS energetic yield. The consequences on cellular homeostasis could be potentially deleterious, since ATP synthesis decline is one of the major pathomechanism involved in the CI deficiency-related phenotype.

*A. thaliana* NDH-2 naturally coexist with CI: their activity is stimulated when electron flux through mitochondrial OXPHOS is slowed down, most likely depending on their intrinsic enzymatic properties [15–18, 33]. Hence, they represent valuable candidates for complementing a defective CI activity without competing with it. AtNDA2, and AtNDB4, in particular, show a substrate preference for NADH over NADPH and their catalytic activity is Ca^2+^ independent, similarly to CI. AtNDA2 is typically detected in the mitochondrial inner membrane, facing the matrix [15, 29], but there is evidence of an additional peroxisomal location [34]. AtNDB4 instead faces the intermembrane space. To target these proteins specifically to the mitochondrial matrix of mammalian fibroblasts, the plant-specific mitochondrial targeting sequence (MTS) was replaced with the human MTS.

AtNDA2, and AtNDB4 were both able to rescue the biochemical defect when expressed in CI-deficient cells, as indicated by increased respiration determined by oxygen consumption determination and lowered SOD activity, a surrogate of ROS production. Functional expression of plant NDH-2 was further indicated by complementation of defective CI on spectrophotometric assays, since both enzymes were able to re-establish NADH:quinone oxidoreductase activity close to control values. Besides, they did not affect cell growth both in standard culture conditions and in the case of glucose deprivation, when cells are forced to switch on OXPHOS for energy production. This observation suggested a lack of competition with CI in standard culture conditions. However, the absence of any apparent effect on cellular growth could be also due to inadequate strength of competition or low level of NDA2 in proportion to CI preventing from detection of such a competition.

We decided to focus on AtNDA2, which represents the most promising candidate to replace CI in deficient cells based on its location and activity profile within plant mitochondria [16, 18, 35] To verify this possibility, we expressed AtNDA2 in human control fibroblasts and evaluated the effect on the ADP phosphorylation levels under different respiratory substrates. Using an NADH related substrate (glutamate/malate), the P/O ratio of transfected cells was lowered by half when compared to control cells. This indicates that AtNDA2 is active when expressed in control cells and competes with CI for electron transfer from NADH to quinone. Additionally, rotenone resistant NADH oxidase activity in AtNDA2-control cells has an apparent K_M_ of 9.7 μM for NADH which is slightly over 3-fold higher than affinity of CI for NADH evaluated in control cells (2.7 μM). Hence, in our experimental model AtNDA2 and CI affinity for the same substrate seem to fall within a similar order of magnitude, thus supporting the existence of a competition in human cells.

However, there are few important limitations linked to this measurement that need to be considered.

Previous studies on plant mitochondria had calculated rotenone-resistant NADH oxidase activity of the inner membrane to be up to 10-fold higher than K_M_ of CI [17, 29, 36], although other authors later reported a considerably lower value of 13.9 μM [37], which is closer to our results.

Likewise, reported apparent K_M_ values of CI for NADH are quite heterogeneous, ranging from 2 μM to 20 μM [38–41].

There are few important considerations to explain the observed intergroup variability. First of all, the development of a specific method to evaluate kinetic properties of CI has notoriously been a recurrent challenge for researchers [23, 42, 43].

Besides this, we should also consider methodological heterogeneity (e. g. sample preparation, category of quinone-analogues employed as electron acceptors, difficulty to accurately estimate enzymatic activity when dealing with extremely low concentration of the substrate etc). Indeed, kinetic properties of these enzymes have been mainly estimated on isolated mitochondria/submitochondrial preparations and on different cell lineages, while we studied permeabilised cell preparations, which are unavoidably contaminated to some extent by soluble NADH dehydrogenases activities. Moreover, AtNDA2 compartmentation within the inner surface of mitochondrial membrane or its association to a supramolecular complex (enzyme malic/specific quinone pool/AOXs) under native conditions, could contribute to its distinctive kinetic properties for NADH and to prevent competition with CI, thus ensuring AtNDA2 activity only in specific physiological circumstances [44].

Most reasonably, the apparent competition with the endogenous OXPHOS system could depend on the plant enzyme concentration within human mitochondria. In our experimental system we used a strong promoter and reached an overall AtNDA2 RNA expression level (25 FPKM) falling in the range of complex I subunit expression (median 29 FPKM). Interpreting these data as a rough measure to approximate the protein levels (unfortunately, we lack information about the post-transcriptional effects for both, AtNDA2 and complex I), would indicate quite high AtNDA2 levels when compared to *A. thaliana*, where AtNDA2 expression has been reported to be up to 10-fold lower than complex I [45]. This very high level of plant enzyme could thus result in the observed competition between AtNDA2 and complex I in our cellular model.

Therefore, our data suggest to test experimental systems exhibiting lower expression levels of NDH-2 for further studies.

## Conclusions

In conclusion, we showed that transfection of plant NDH-2 was able to rescue CI defect in vitro. However, AtNDA2, the most promising candidate based on its properties in plants, exhibits competing activity with human CI when expressed at high levels, thus raising concerns which need to be considered in case of its application to human therapy. In cells with substantial CI activity, a balance in terms of energy production and metabolic dysfunctions needs to be determined, in which the gain of additional NADH oxidation is more beneficial than the reduced ATP production through competition with CI. If not controlled, consequences of this uncoupling effect are unpredictable in vivo, and risk to be deleterious in affected patients. A substantial amount of translational work still has to be done in the near future, from genetic manipulation of the transfected plant product to possible modification of its enzymatic properties, to the generation of an animal model to test its effects in vivo.

Nevertheless, we have moved an important step towards a deeper comprehension of potential advantages and drawbacks of trans-kingdom replacing therapy for respiratory chain defects.

## Additional file

Additional file 1: Supplementary methods. Supplementary results. **Table S1.** Expression level of AtNDA2, AtNDB4, and NADH:quinone oxidoreductase (CI) subunits in various compartment of *A.thaliana. (DOCX 105 kb)*

## Data Availability

The datasets used and/or analysed during the current study are available from the corresponding author on reasonable request.
