# Supplementary material for: Arabidopsis thaliana alternative dehydrogenases: a potential therapy for mitochondrial complex I deficiency? Perspectives and pitfalls
Source: Orphanet J Rare Dis. 2019 Oct 29;14:236. doi: 10.1186/s13023-019-1185-3 (PMC6821020; doi:10.1186/s13023-019-1185-3)
Supplement: Supplementary file 1 — Supplementary methods. Supplementary results. Table S1. Expression level of AtNDA2, AtNDB4, and NADH:quinone oxidoreductase (CI) subunits in various compartment of A.thaliana. (DOCX 105 kb) [file 13023_2019_1185_MOESM1_ESM.docx]

**Supplementary Methods:**

**Cloning ScNDI1, AtNDA1, AtNDA2, and AtNDB4 in pLenti 6.3**

The full length *ScNDI1* ORF was amplified using yeast DNA from the strain YPH499 and the following primers: F 5’cacgATGCTATCGAAGAATTTGTATAGTAACAAGAG3’; R: 5’ CTATAATCCTTTAAAAAAGTCTCTTTTGAAAAATGCTAA3’. To the full length *ScNDI1* ORF the mitochondrial targeting sequence of the human *COXVIII* gene and a 6xHis tag were inserted at the N-terminal end of *ScNDI1* using the forward primer F: 5’cacgATGTCCGTCCTGACGCCGCTGCTGCTGCGGGGCTTGACAGGCTCGGCCCGGCGGCTCCCAGTGCCGCGCGCCAAGATCCATTCGTTGCATCATCACCATCACCAT3’. The reverse primer was designed to keep the natural ScNDI1 stop codon R: 5’CTATAATCCTTTAAAAAAGTCTCTTTTGAAAAATGCTAA3’.

The *AtNDA1, AtNDA2* and *AtNDB4* ORFs were amplified using plasmid templates encoding recombinant *A. thaliana* polypeptides truncated at the N-terminal end and carrying an N-terminal T7 tag. The T7 tag was fused to amino acid residue 69 in NDA1, 67 in NDA2 and 59 in NDB4[1]. At the N-terminal end of *AtNDA1*, *AtNDA2* and *AtNDB4* was inserted the mitochondrial targeting sequence from the human *COXVIII* gene using the forward primer F: 5’cacgATGTCCGTCCTGACGCCGCTGCTGCTGCGGGGCTTGACAGGCTCGGCCCGGCGGCTCCCAGTGCCGCGCGCCAAGATCCATTCGTTGATGGCTAGCATGACTGGTGGACAGCAAATGGGT3’. The reverse primers were designed to keep the natural *AtNDA1*, *AtNDA2* and *AtNDB4* stop codons (AtNDA1_R: 5’TCAGATTCGGCTAATGTCCCGACCA3’, AtNDA2_R: 5’TTAGATACGGCTAATGTCACGACCAAAAA3’, AtNDB4_R: 5’TTAGATGCTGCTAGAGTCACGGC3’). The PCR was done using the Platinum™ Taq DNA Polymerase High Fidelity (Invitrogen) and products cloned with the pLenti6.3/V5-TOPO TA Cloning Kit (Thermo Fisher Scientific) following the manufacturer’s instructions.

The final plasmids were analyzed by DNA sequencing and were found consistent with available cDNAs annotations. These were: ScNDI1, NM_001182483.1; Atnda1, NM_100592.5; Atnda2, NM_128553.3; Atndb4, NM_127645.4. The plasmids were denoted ScNDI1_pLenti, MTS_ScNDI1_pLenti, AtNDA1_pLenti, AtNDA2_pLenti, AtNDB4_pLenti.

**Outline of the generated recombinant NDH-2.** The alternative dehydrogenase from yeast was produced in two forms. A form retaining the natural yeast mitochondrial targeting sequence (MTS) expected to produce a protein of 57.3 kDa, and a second form carrying the additional MTS from the human COXVIII sub-unit and a His-tag, expected to produce a 61.2 kDa protein. The Arabidopsis NDH-2, were cloned using only the mature forms of NDA1, NDA2 and NDB4, in which the plant MTS was replaced by the human COXVIII MTS and a T7-tag. The expected M.W. of the recombinant proteins was 53.8 kDa, 54 kDa and 63.7 kDa, respectively.

Mature_At_NDB4

Mature_At_NDA1

Mature_At_NDA2

Sc_NDI1

MTS

Sc_NDI1

MTS

MTS

COX VII

MTS

COX VII

MTS

COX VII

MTS

COX VII

His

T7

T7

T7

**Western Blotting**

Total cellular lysates were homogenized in RIPA buffer in presence of protease inhibitors Calbiochem). Insoluble material was removed by centrifuging cellular lysates at 15000xg for 15 minutes at 4°C. Following protein quantification (Bradford), 15 µg of each sample were taken. Samples were denatured at 70°C for 10 minutes in presence of Laemmli buffer, using DTT as reducing agent. Samples were loaded on a 10% denaturing pre-cast gel (Cat. No. 58714, PAGEr^TM^ EX Gels, Lonza) and transferred on a PVDF membrane using a semi-dry protocol according to the manufactory instructions (ProSieve^TM^ EX Western Blot Transfer Buffer, Lonza).

**Supplementary Results:**

**Expression of yeast NDI1 recombinant proteins in mammalian cells**

The expression of the yeast NDI1 proteins, with the natural MTS and with the additional human MTS, in fibroblasts from a healthy control and from mitochondrial patients carrying pathogenic variants in ACAD9 and NDUFS4 sub-units, was evaluated by western blotting. Proteins were extracted from samples before transduction (NDHF, 35834, 79787, 87971) and after transduction (NDHF-T-, 35834-T-, 79787-T-, 87971-T-). Transduced cell lines expressed equally well both transgenic variants of ScNDI1, with and without the human MTS. As expected, not transduced lines did no express the recombinant protein. ScNDI1 expression was normalized to GAPDH.

**
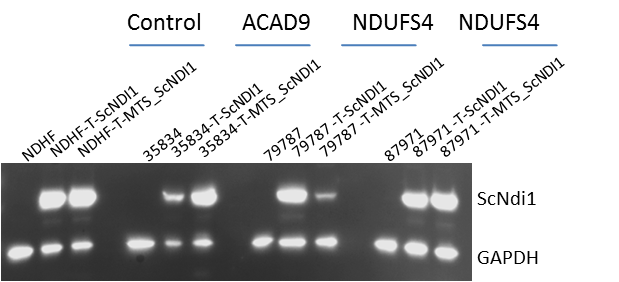
**

**Table S1:** Expression level of AtNDA2, AtNDB4, and NADH: quinone oxidoreductase (CI) subunits in various compartment of *A. thaliana*.

| Gene Name | leaf (FPKM) | flower (FPKM) | fruit (FPKM) | root (FPKM) |
| --- | --- | --- | --- | --- |
| NDA2 | **5** | **8** | **7** | **10** |
| NDB4 | 0.1 | 0.4 | 0.1 | 0.1 |
| GAMMACA2 | 30 | 53 | 43 | 60 |
| AT1G79010 | 65 | 96 | 79 | 109 |
| AT2G02050 | 65 | 116 | 95 | 114 |
| AT2G20360 | 72 | 116 | 98 | 140 |
| AT2G33220 | 48 | 51 | 59 | 54 |
| AT2G47690 | 21 | 34 | 35 | 52 |
| AT3G03070 | 79 | 108 | 98 | 114 |
| AT3G08610 | 121 | 162 | 148 | 162 |
| AT3G18410 | 30 | 49 | 52 | 66 |
| GAMMACAL2 | 30 | 43 | 33 | 70 |
| AT4G02580 | 72 | 112 | 94 | 129 |
| AT4G16450 | 63 | 92 | 91 | 120 |
| AT4G20150 | 96 | 129 | 115 | 154 |
| AT5G11770 | 70 | 87 | 90 | 131 |
| AT5G18800 | 41 | 73 | 57 | 94 |
| AT5G47570 | 65 | 78 | 72 | 82 |
| AT5G47890 | 56 | 72 | 66 | 102 |
| AT5G52840 | 70 | 85 | 84 | 102 |
| GAMMA CAL1 | 32 | 48 | 44 | 61 |
| GAMMA CA3 | 39 | 63 | 52 | 96 |
| NAD5C | 0,6 | 2 | 3 | 2 |
| NAD2A | 0.2 | 0.6 | 0.7 | 0.4 |
| NAD1C | 0.6 | 2 | 2 | 2 |
| NAD4 | 0.3 | 1 | 2 | 1 |
|  |  |  |  |  |
| **Median CI FPKM** | **52** | **72,5** | **62,5** | **95** |

Expression levels are expressed in FPKM. Values have been extracted by the public database (https://www.ebi.ac.uk/gxa/home) and refer to *A. thaliana* growing under basal condition. In gray are labelled the NADH: quinone oxidoreductase subunits, in green the two NDH-2 genes.

**Reference:**

1. Geisler DA, Broselid C, Hederstedt L, Rasmusson AG. Ca2+-binding and Ca2+-independent respiratory NADH and NADPH dehydrogenases of Arabidopsis thaliana. J Biol Chem. 2007;282(39):28455-64.
